# Effectiveness of a multi-component intervention including pictorial warnings to reduce sugar-sweetened beverage consumption - a randomized controlled trial

**DOI:** 10.1186/s12966-025-01800-0

**Published:** 2025-08-30

**Authors:** Kumar Guru Mishra, Aliya Afreen, Nabnita Patnaik

**Affiliations:** 1 Department of Community Medicine, Apollo Institute of Medical Sciences and Research, Hyderabad, Telangana India; 2 Department of Obstetrics and Gynecology, AIIMS Bibinagar, Hyderabad, Telangana India

**Keywords:** Sugar-sweetened beverages, Pictorial health warnings, Consumer behavior, Urban health, Health promotion

## Abstract

**Introduction:**

Sugar-sweetened beverages (SSBs) are a significant source of added sugars in the Indian diet, contributing to adverse health outcomes such as obesity, type 2 diabetes, and dental caries. Multi-component interventions (MCIs) have shown promise in reducing the consumption of harmful products like tobacco. This study assessed the effectiveness of a multi-component intervention—comprising Pictorial Health Warnings (PHWs), educational pamphlets, and targeted counseling—in reducing parental purchases of SSBs for children.

**Methods:**

A randomized stepped-wedge controlled trial was conducted across three urban slum sectors in Hyderabad. A total of 60 parents were recruited, with data collected over ten weeks. MCIs were implemented on SSBs at general stores, and outcomes were measured by comparing SSB purchase frequency, risk perception, and parental attitudes before and after the intervention.

**Results:**

The implementation of MCIs led to a significant reduction in SSB purchases, with 25% of parents buying SSBs post-intervention compared to 55% pre-intervention (*p* = 0.002). Parents also purchased SSBs with fewer calories post-intervention (45 kcal vs. 92 kcal, *p* < 0.001). Additionally, MCIs increased awareness of SSB-related harms (d = 2.19, *p* < 0.001) and strengthened negative emotional responses (d = 2.08, *p* < 0.001). No significant differences were observed in the appeal or perceived tastiness of SSBs (*p* > 0.05).

**Conclusion:**

MCIs on SSBs effectively reduced purchase frequency and calorie intake among parents in urban slum settings. The findings support the potential for MCIs to be a feasible public health intervention to reduce SSB consumption in similar socio-economic contexts.

**Trial registration:**

CTRI/2024/08/072220 dated 09/08/2024.

**Supplementary Information:**

The online version contains supplementary material available at 10.1186/s12966-025-01800-0.

## Introduction

Sugar-sweetened beverages (SSBs) have emerged as a significant source of added sugar in the Indian diet. These beverages, containing various forms of added sugars such as dextrose, fructose, glucose, high fructose sugar syrup, maltose, molasses, and raw sugar, offer minimal nutritional value and cannot serve as meal replacements. Unlike solid foods, liquid sugars may provide a rapid energy boost but fail to induce comparable levels of satiety [1].

The consumption of SSBs is associated with numerous adverse health outcomes, including obesity, type 2 diabetes, dental caries, cardiac damage, and fatty liver disease [2, 3]. Moreover, the highly palatable nature of sugar can lead to addictive behaviour, and a sugar-rich diet has been linked to mental health issues such as depression, anxiety, and impulsive hunger [4].

Recognizing these concerns, the Nutrition Chapter of the Indian Academy of Pediatrics (IAP) has issued recommendations regarding fruit juice and SSB consumption for children and adolescents. They advise prioritizing regional and seasonal whole fruits over fruit juices and suggest strict limitations on SSB intake based on age groups [5].

The influence of media, particularly child-targeted television commercials, has been correlated with increased sugar consumption. However, as a significant portion of a child’s daily calorie intake occurs at home, positive parental modeling can play a crucial role in shaping children’s snack preferences and reducing SSB consumption [6].

Previous research on tobacco control has demonstrated the effectiveness of plain packaging with Pictorial Health Warnings (PHWs) in reducing product appeal, increasing warning noticeability, garnering public support, and potentially influencing quitting behaviour and preventing initiation [7]. While most existing research on reducing SSB consumption has evaluated the use of PHWs or nutritional labeling in isolation, growing evidence suggests that MCIs—those that combine labeling with educational materials or behavioral counseling—can yield greater impact.

Previous studies have demonstrated the effectiveness of pictorial health warnings on SSBs. Grummon et al. [8] in their meta-analysis found that sugary drink warnings significantly reduced selection and purchase intentions across multiple experimental studies. Hall et al. [9] showed that pictorial health warnings on SSBs reduced purchases of sugary drinks for children in a randomized controlled trial. Building on these findings, emerging evidence suggests that multi-component interventions may yield greater impact than single interventions alone [10–13].

The success of PHWs in tobacco control, where graphic warnings have significantly impacted consumer behavior and smoking cessation [14], provides a foundation for applying similar strategies to SSB control. Research in tobacco control demonstrates that comprehensive approaches combining multiple intervention elements can enhance effectiveness [15, 16].

However, there is a lack of field-based trials evaluating integrated interventions that combine PHWs with pamphlets and personalized counseling, particularly in low-income or low-literacy settings like urban slums in India. Our study addresses this gap by testing a MCIs combining PHWs, pamphlet distribution, and individualized counseling to reduce SSB purchases by parents.

While beverage bottles typically contain nutritional and ingredient information, this data is often obscured or incomprehensible to the general population due to small font sizes or the use of technical language. MCIs offer a concise explanation of the risks associated with excess consumption and may strongly influence drink selection by eliciting emotional or adverse reactions. Moreover, MCIs have the potential to better reach vulnerable populations and those with low socioeconomic status [17]. A stepped-wedge randomized controlled design was selected due to its feasibility in resource-constrained settings where withholding the intervention permanently from a control group would be unethical. Given the urgent need for population-level efforts to reduce SSB intake, enforcing MCIs on SSBs could prove to be an effective strategy. This study employed a stepped-wedge randomized control trial across three urban slum sectors in Hyderabad to analyze changes in parents’ SSB purchase frequency for their children following a MCI intervention.

The primary aim of this study was to measure the change in the frequency of parents’ purchase of SSBs for their children following a two-week MCI intervention. Additionally, we sought to determine:

1. The change in the overall perception of parents towards the consumption of SSBs by children.
2. The change in risk-perception of parents towards consumption of SSBs by children.
3. The change in the consumption attitude of parents towards SSBs.
4. The change in parents’ intention to limit the consumption of SSBs by their children.

By addressing these objectives, this research aims to contribute to the reduction of life-threatening conditions associated with SSB consumption and improve dietary habits among children. Through raising awareness about the risks of SSBs, particularly among parents, we hope to foster a more health-conscious approach to beverage choices in families residing in urban slum areas.

## Materials and methods

### Study design and setting

It was a randomized stepped-wedge controlled trial design conducted across three urban slum sectors in Hyderabad between September 2024 and December 2024, after obtaining ethical clearance from the Institutional Ethics Committee of Apollo Hospitals, Hyderabad (EC/NEW/INST/1527/2023/09/125), and registration with the Clinical Trials Registry, India (CTRI/2024/08/072220 dated 09/08/2024). The selected urban slums are characterized by high population density, lower literacy rates (~ 60%), and average household monthly incomes below ₹10,000. All participants provided written informed consent.

**Fig. 1:**
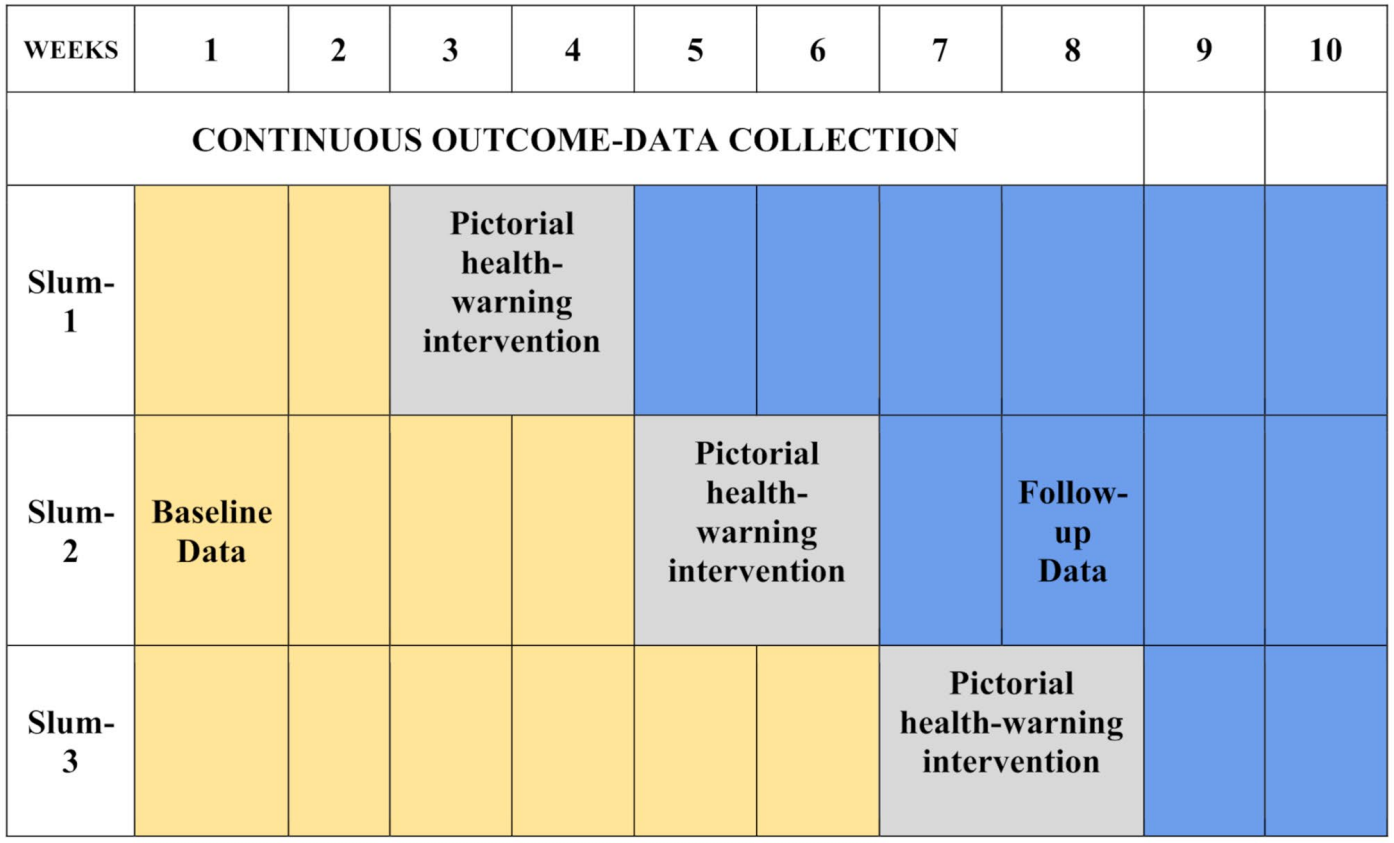
Trial design and implementation of intervention & data collection over 10-weeks

The stepped-wedge design allowed all clusters to eventually receive the intervention while enabling within- and between-cluster comparisons, making it an ethically suitable and pragmatic choice for this setting. Repeated cross-sectional outcome data were collected weekly for ten weeks.(Fig. 1) Baseline data (control phase) were gathered from each slum sector two weeks prior to the intervention’s commencement. A two-week MCIs was implemented in a stepped manner across all three slum sectors at two-week intervals. Follow-up data collection continued for two weeks after the intervention’s completion in the third sector. The trial outcome was determined by comparing overall perception and behavioral changes towards SSBs among parents between baseline and follow-up periods across all three sectors combined. Strict adherence to ethical and scientific committee guidelines was maintained throughout the study.

### Randomization and blinding

The order of intervention implementation was randomly allocated across the three slum sectors using a computerized random number generator. Allocation was undertaken simultaneously for all sectors. Participants providing outcome data were blinded to the experimental nature of the intervention implementation across slums. However, due to the nature of the intervention, survey personnel were aware of when their service was in the intervention period.

### Participant selection and recruitment

Local Accredited Social Health Activists (ASHAs) developed the sampling frame by listing households with at least one child under 10 years of age, based on government records and routine home visits. This approach ensured that the sample was representative of the typical demographic structure of the urban slum population. Inclusion criteria encompassed parents or guardians of at least one child under 10 years of age who understood the local languages (Hindi, Telugu) or English. Individuals from households with previous study participants were excluded. Eligible participants were identified from lists provided by local health workers. General store owners marked participants who made beverage purchases. Weekly random sampling of eligible participants who had visited a general store in the past week was conducted using a computerized random number generator. Selected participants received an information statement explaining the survey’s purpose, and written Informed consent was obtained from all participants prior to their inclusion in the study. For participants under the age of 16, informed consent was obtained from their parents or legal guardians. Additionally, for illiterate participants, consent was obtained through their legally authorized representatives or guardians in accordance with ethical guidelines.

Participants retained the right to decline participation or withdraw at any point during the study.

### Intervention

The MCI was implemented in all general stores across the three slums. Written informed consent was obtained from all store owners prior to the intervention. They were briefed about the public health purpose of the study, assured that no financial burden would be placed on them, and encouraged to participate as a contribution to community health promotion. No monetary incentives were offered.

**Fig. 2:**
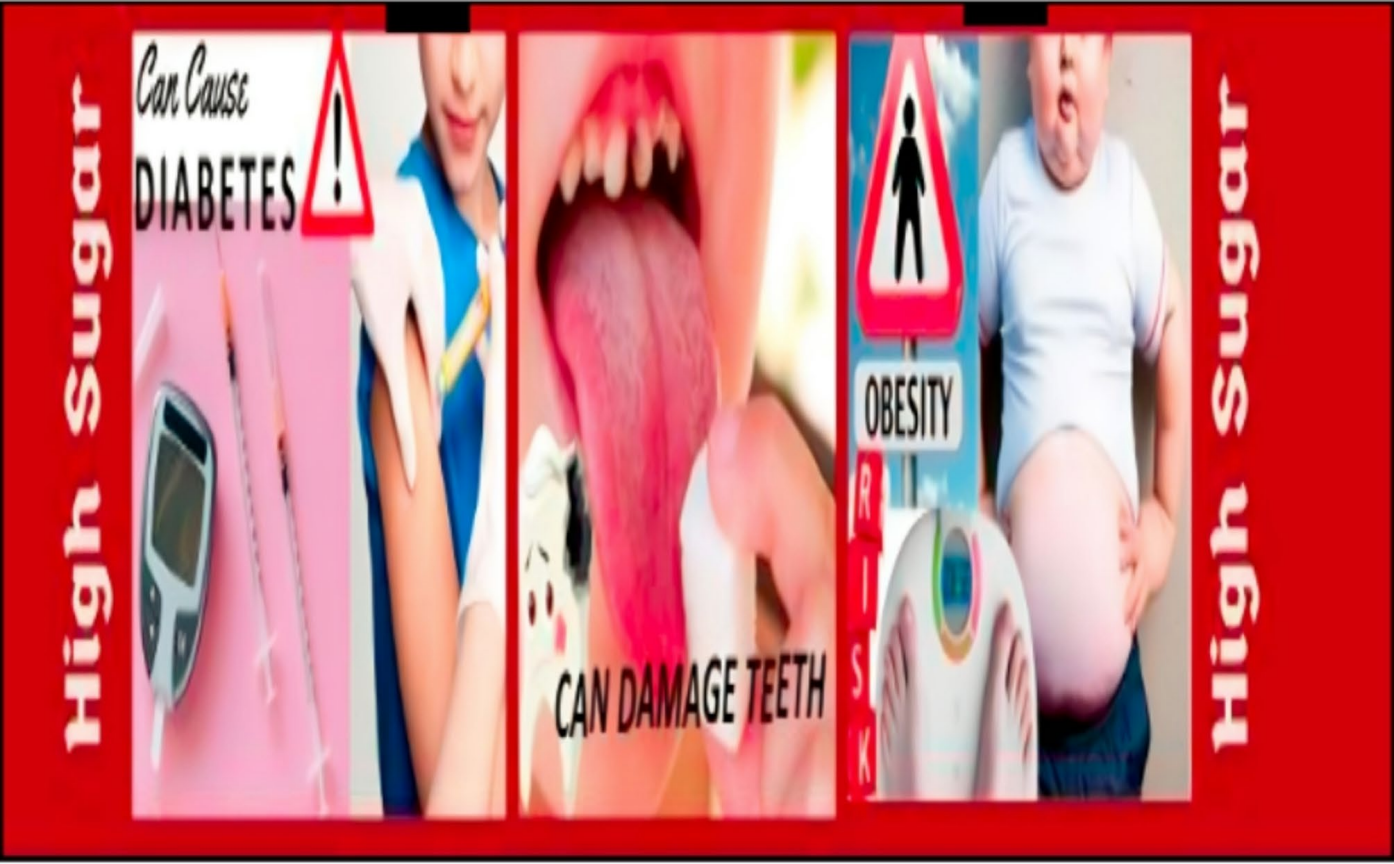
Shows the PHW label showing type 2 diabetes, tooth decay, and obesity

**Fig. 3:**
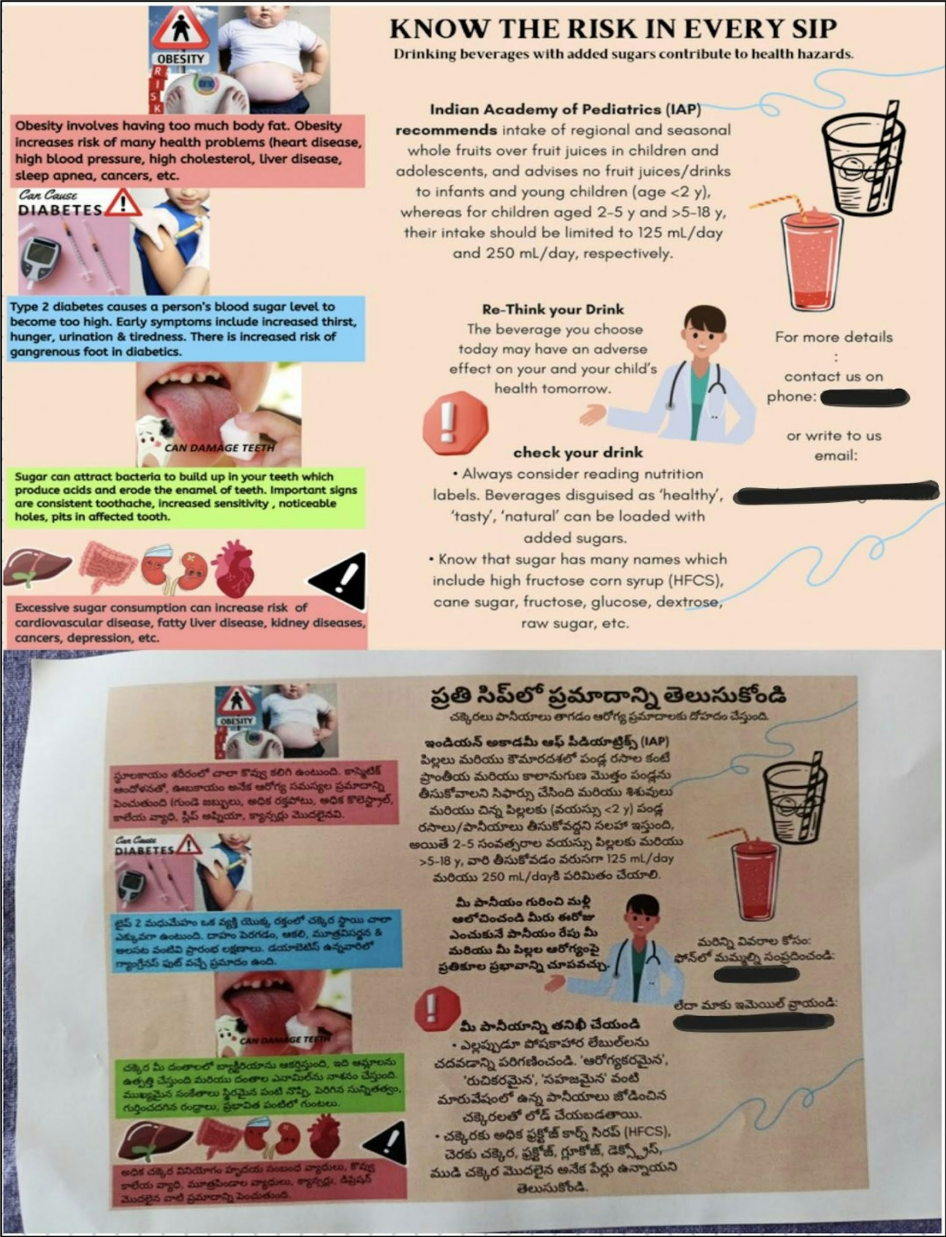
Pamphlet explaining the risk of overconsumption of SSBs. (In english and local language)

Participants visiting these stores from the intervention’s commencement received the intervention. PHW labels depicting adverse effects of type 2 diabetes, tooth decay, and obesity were added to existing labels on beverage bottles. (Fig. 2) Labels were placed on soda/cold drinks, fruit drinks, flavored milk, and sports drinks, reflecting the primary categories of SSBs sold in local general stores. Additionally, pamphlets containing detailed explanations in English and the local language were distributed to participants along with purchased SSBs.(Fig. 3).

In this study, SSBs were defined as beverages containing added sugars, including soda/cold drinks, fruit drinks, flavored milk, and sports drinks, reflecting the main types sold in local stores. Although sports drink consumption was relatively less frequent in this population, they were included to ensure a comprehensive assessment of available sugary beverages.

The intervention was multi-component in nature, combining pictorial health warning labels, educational pamphlets, and one-on-one counseling for parents of children with moderate to high SSB intake.

### Intervention components

The intervention comprised three key components— PHWs, educational pamphlets, and one-on-one counseling—each designed using behavior change theories and tailored to the local context.

### Pictorial health warning labels (PHWs)

The PHW labels were designed following evidence from tobacco control research [18] and health communication theory [19]. Each label measured 5 cm × 8 cm and was applied to cover 50% of the front panel of beverage containers. The warnings included high-contrast images illustrating health consequences of excessive sugar consumption, such as complications of type 2 diabetes, severe dental caries, and childhood obesity. These visuals were accompanied by brief text messages in Hindi, Telugu, and English. The design employed principles of fear appeal theory to enhance emotional engagement and message salience by using vivid, attention-grabbing imagery [20].

### Educational pamphlets

The educational pamphlets were developed using the Health Belief Model [21] and social cognitive theory [22] to support behavior change. They included visual illustrations of sugar content in popular SSBs (expressed in teaspoons), scientific information on associated health risks, and practical guidance on choosing healthier alternatives. Additional content included tips on reading nutrition labels, interpreting ingredient lists, and understanding age-appropriate serving size recommendations, aligned with IAP guidelines. Pamphlets were visually engaging, designed with culturally appropriate images, and translated into Hindi and Telugu by certified translators. Their content was validated by a panel of three public health experts and pre-tested with 10 parents from a similar demographic background to ensure relevance and clarity.

### One-on-one counseling protocol

Personalized counseling was delivered by trained health educators using a standardized approach based on motivational interviewing techniques [23] and the Transtheoretical Model of behavior change [24]. Two educators with undergraduate qualifications in nutrition or public health underwent 16 h of training. The training covered key areas such as motivational interviewing, nutritional counseling related to SSBs, cultural sensitivity in communication, and adherence to standardized counseling protocols.

Each participant received two semi-structured counseling sessions during the intervention period (Week 1 and Week 2), with each session lasting 15–20 min. Sessions were conducted in a private space within or near general stores. The counseling process was structured into three phases: (a) Assessment Phase (5 min): Counselors assessed current SSB consumption patterns in the household, parental beliefs and attitudes about SSBs, and barriers to behavior change. (b) Education Phase (8–10 min): Personalized feedback was provided on the child’s SSB intake, followed by a discussion on health risks relevant to the family. The health educator reviewed the pamphlet, reinforcing key messages. (c) Goal-Setting Phase (5 min): The session concluded with a collaborative goal-setting exercise where the counselor and parent developed realistic reduction targets, identified practical strategies, and anticipated potential barriers and solutions.

### Quality assurance

All counseling sessions were audio-recorded (with informed consent) to monitor fidelity to the protocol. Weekly supervision meetings with the principal investigator were held to address implementation issues. Additionally, 20% of randomly selected session recordings were reviewed to ensure adherence to the counseling framework.

### Data collection and assessment

Outcome assessment was conducted through a validated questionnaires used by Hall et al. [9] in a similar research trial, with risk scores (low/medium/high) calculated based on responses. Participants completed questionnaires in person weekly. Risk scores indicated the risk level for adverse child health behaviors based on frequency and amount of SSB purchases and consumption. All parents, regardless of risk scores, received information about adverse health consequences, consumption frequency, and the significance of nutritional and ingredient information for SSBs. Parents of children with moderate or high SSB consumption received counseling on implementing positive lifestyle changes, monitoring beverage consumption, and opting for healthier drink choices. Counseling was conducted during the intervention period, concurrent with weekly data collection, and was not delayed until study completion. Label noticing relates to message salience, while anticipated social interactions refer to subjective norms from the Theory of Planned Behavior.

### Control phase

In each slum sector, pre-intervention data served as the control. No specific study-related information was provided during the control phase. The research team controlled the initiation and delivery of intervention elements. Intervention strategies were inaccessible to participants during the baseline (control) phase. Potential for contamination due to staff movement between slums was considered limited due to the structural and systemic nature of the implementation strategies. The three slums were geographically separated by approximately 3–5 km, minimizing the risk of contamination between clusters. Information on participant movement between selected slums was collected throughout the study.

### Outcomes

**Fig. 4:**
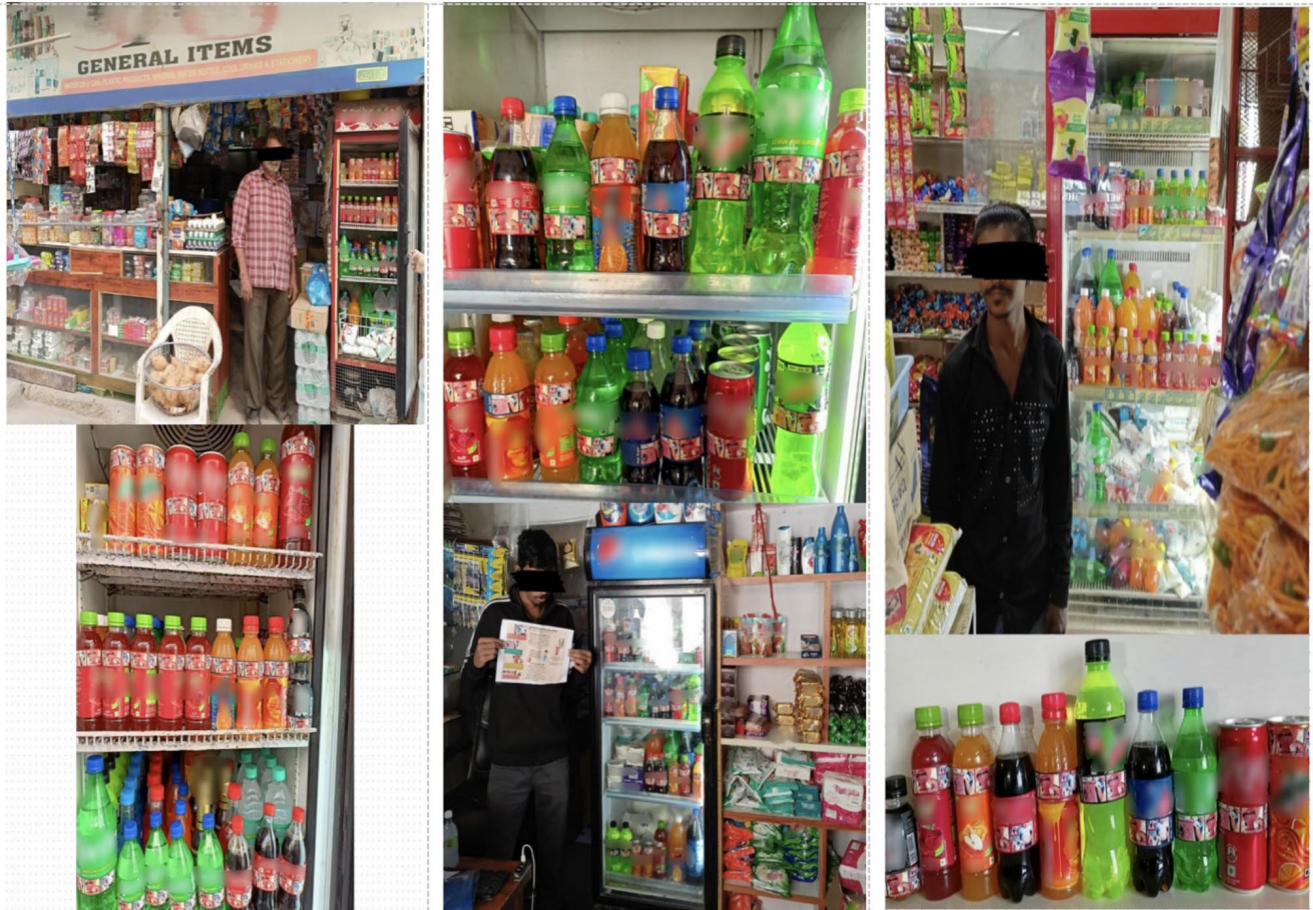
Photographs of general stores showing PHW label intervention. Brand names of beverages are blurred to prevent identity. ^*^All individuals appearing in photographs in Fig. 4 provided written consent for their images to be published. Their faces have been masked to maintain confidentiality

**Fig. 5:**
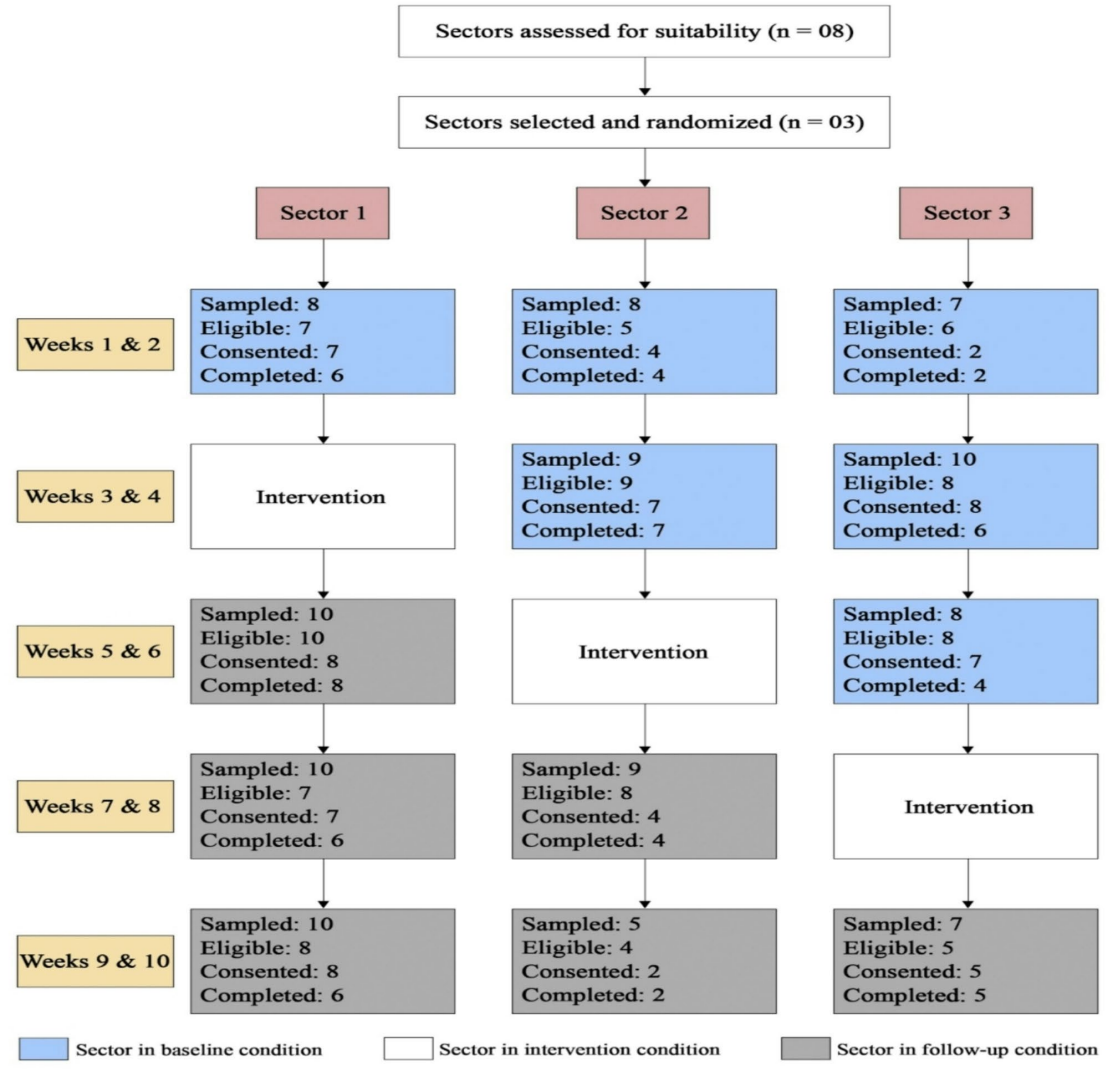
Flow diagram depicting participant numbers throughout the trial

Primary outcomes included changes in overall perception pre- and post-intervention, purchase frequency, risk perception, consumption attitude, and parents’ intention to limit SSB consumption for their children. The secondary outcome was participants’ acceptance of PHW labels on SSBs. (Figures 4 and 5)

### Data management and confidentiality

Research data were securely stored and accessible only to primary researchers and statisticians. Participant confidentiality was strictly maintained throughout the study. All individuals appearing in photographs in Fig. 4 provided written consent for their images to be published and their faces have been masked to protect privacy.

### Sample size calculation

A sample size of 60 participants was determined to provide 80% power to detect an absolute increase of 15% in perception during the intervention period (based on a conservative 50% estimate of baseline perception prevalence) at a significance level of *p* = 0.05, assuming an intra-cluster correlation of 0.01. With approximately 50 eligible participants per month, a weekly sample of 12 participants and an 80% survey completion rate (*n* = 10) was projected to yield the required number of participants per week.

### Statistical analysis

Baseline and follow-up primary outcomes data were analyzed using a logistic mixed model to detect changes over time in the reported receipt of MCIs. Descriptive statistics were used to report baseline data. Statistical significance was set at *p* < 0.05. SPSS Version 26 was used for all statistical analyses. *P*-values and 95% confidence intervals for hypothesis testing were derived using independent z-tests comparing proportions for dichotomous outcomes and independent samples t-tests for Likert-style items and total calories from SSBs. Standardized effect sizes (Cohen’s d) were calculated to standardize the effects.

### Moderation analysis

The potential moderating effects of participant and child characteristics on the impact of warning labels were explored by including interaction terms between the study arm and each characteristic in logistic regression models. These characteristics included parents’ age, gender, educational levels, monthly household income, Nutrition Facts Panel use, frequency of needing help reading medical information, age and gender of the child the parent shopped for, and the child’s SSB consumption. Separate logistic regression models were run for each moderator, regressing the trial arm, the moderator, and their interaction on the primary outcome, examining the statistical significance of the moderator term.

## Results

### Participants characteristics

The study recruited participants between October and December 2023. Of 101 parents initially selected, 85 met eligibility criteria, 69 provided consent, and 60 completed the interview questionnaire. The participant demographic profile revealed a mean parental age of 36 years, with 65% women and 35% men. Approximately two-thirds of parents reported an annual household income below ₹70,000, and 60% had educational attainment of high school diploma or less. Regarding children’s age groups, 43% of parents shopped for children aged 2–5 years, while 53% shopped for those aged 5–10 years (Table 1).

**Table 1:** Baseline (pre-intervention) socio-demographic characteristics of study participants (*n* = 60)

Characteristics	N (%)
**Age (years)**	
18–29	17 (28.33)
30–39	33 (55.00)
40–49	4 (6.66)
≥ 50	6 (10.00)
**Age, Mean ± SD**	36.34 ± 3.65
**Gender**	
Male	21 (35.00)
Female	39 (65.00)
**Education**	
Illiterate	3 (11.84)
Primary School Certificate	2 (3.33)
Middle School Certificate	18 (30.00)
High School Certificate	14 (23.33)
Intermediate	5 (8.33)
Graduate/ Post Graduate	15 (25.00)
Profession/ Honors	3 (5.00)
**Monthly Household income**	
≤ ₹9,226	14 (23.33)
₹9,232 – ₹27,648	16 (26.67)
₹46,094 – ₹68,961	15 (25.00)
₹68,967 – ₹92,185	10 (16.67)
₹92,191 – ₹1,84,370	3 (5.00)
≥ ₹1,84,376	2 (3.33)
**Number of people in the household, Mean ± SD**	3.4 ± 1.6
**Nutrition Facts Panel use**	
Never	19 (31.67)
Rarely	25 (41.67)
Sometimes	10 (16.67)
Often	5 (8.33)
All the time	1 (1.67)
**Frequency of needing help reading medical information**	
Never	9 (15.00)
Sometimes	30 (50.00)
Often	13 (21.66)
Always	8 (13.33)
**Age of child the parent shopped for (years)**	
≤ 2	2 (3.33)
2–5	26 (43.33)
6–10	32 (53.33)
**Gender of the child the parent shopped for**	
Boy	41 (68.33)
Girl	19 (31.67)
**Child consumed SSBs 1/week or more over past 30 days (not mutually exclusive)**	
Soda/ Cold Drinks	47 (78.33)
Fruit Drink	42 (70.00)
Flavored Milk	34 (56.67)
Sports drink	16 (26.67)

### Primary findings: impact on SSB purchasing and perceptions

The implementation of MCIs on SSBs demonstrated a significant impact on parental purchasing behavior. Post-intervention, only 25% of parents purchased SSBs for their children, compared to 55% in the pre-intervention period (*p* = 0.002), representing a 30% reduction in SSB purchases. Additionally, parents exposed to MCIs purchased SSBs with fewer calories (45 kcal) compared to the control group (92 kcal) (d = -0.34, *p* < 0.001) (Table 2).

**Table 2:** Impact of pictorial health warnings

Outcome	Pre-intervention (n/mean)	% (SD)	Post-intervention (n/mean)	% (SD)	Difference (95% CI)	*p-value*	Cohen’s d
**Purchase outcomes**							
Purchased a SSB (primary outcome)	33	55.00%	15	25.00%	–30% (–37%, − 22%)	< 0.001*	–0.52
Total calories from SSBs (in kcal)	92.26	(103.21)	45.11	(54.21)	–48.12% (–89.23%, − 24.55%)	< 0.001*	–0.34
**Label Reactions**							
Noticed label	10	16.67%	45	75.00%	58.56% (45.76%, 69.44%)	0.02*	2.05
Felt more in control of healthy eating decisions	25	41.67%	42	70.00%	29.23% (16.29%, 37.11%)	0.03*	1.47
Thinking about the harms of drinking SSBs	1.73	(1.02)	4.27	(0.68)	2.54 (2.07, 2.98)	< 0.001*	2.19
Negative emotional reactions	1.28	(0.76)	3.77	(1.22)	2.49 (1.51, 3.01)	< 0.001*	2.08
Anticipated social interactions	1.76	(0.95)	4.56	(1.04)	2.79 (2.12, 3.48)	0.04*	1.65
Perceived amount of added sugar in SSBs	1.41	(1.08)	4.31	(1.76)	2.89 (2.27, 3.65)	< 0.001*	2.11
**SSB attitudes and intentions**							
Perceived healthfulness of SSBs for child	2.57	(1.88)	0.54	(1.21)	–2.03 (–3.22, − 1.65)	0.04*	–1.21
Appeal of SSBs for child	3.29	(0.92)	2.87	(0.59)	–0.42 (–1.27, 0.17)	0.28	–0.94
Perceived tastiness of SSBs for child	4.18	(1.85)	3.89	(1.29)	–0.29 (–1.37, − 0.75)	0.34	–0.51
Perceived likelihood of child experiencing health problems due to SSBs	1.78	(0.86)	3.11	(1.07)	1.33 (1.04, 1.87)	0.04*	1.06
Injunctive norms to limiting SSBs for child	2.06	(0.85)	4.18	(1.43)	2.12 (1.67, 3.06)	0.003*	2.76
Intentions to give SSBs to child	2.79	(0.77)	0.98	(1.03)	–1.81 (–2.17,–1.01)	0.02*	–1.92

**Fig. 6:**
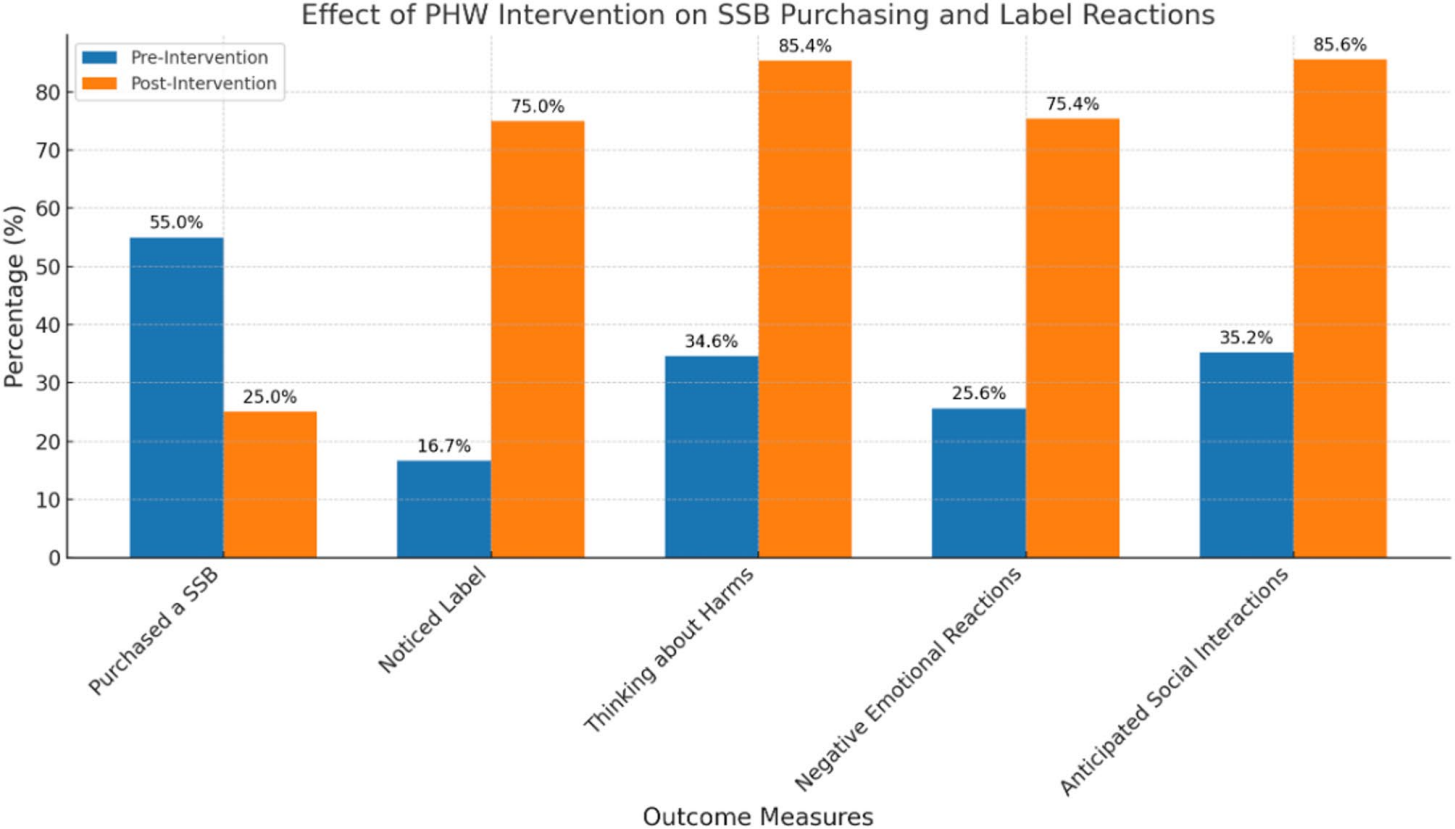
Changes in SSB purchasing and label reactions among parents before and after the PHW intervention. Data labels represent actual percentage values

To visually represent the effect of the intervention, Fig. 6 illustrates the changes in SSB purchasing and key label reactions (noticeability, awareness of harms, emotional responses, and anticipated social interactions) between the pre- and post-intervention periods. The graph highlights a substantial reduction in SSB purchases and significant improvements in health communication outcomes following the implementation of pictorial health warnings.

Exposure to MCIs also elicited stronger label reactions, including increased awareness of SSB-related harms (d = 2.19, *p* < 0.001), heightened negative emotional responses (d = 2.08, *p* < 0.001), and greater anticipated social interactions (d = 1.65, *p* = 0.04). MCIs influenced attitudes and intentions, leading to lower perceived healthfulness of SSBs for children (d = -1.21, *p* = 0.04), stronger injunctive norms to limit SSB consumption (d = 2.76, *p* = 0.003), and reduced intentions to serve SSBs to children (d = -1.92, *p* = 0.02).

The intervention also resulted in significant differences in label noticeability (d = 2.05, *p* = 0.02), and perceived added sugar content (d = 2.11, *p* < 0.001). However, no significant differences were observed in the appeal (*p* = 0.28) or perceived tastiness (*p* = 0.34) of SSBs.

Importantly, 53% of participants reported feeling more empowered to make healthy eating decisions, and 45% expressed support for legislation mandating MCIs on SSBs.

### Moderation analysis: differential impact based on participant characteristics

The effect of MCIs on parental SSB selection remained consistent across various participant characteristics, including age (*p* = 0.58), gender (*p* = 0.93), educational level (*p* = 0.51), monthly household income (*p* = 0.17), Nutrition Facts Panel use (*p* = 0.57), health literacy (*p* = 0.41), child’s age (*p* = 0.45) and gender (*p* = 0.65), and child’s SSB consumption habits (*p* = 0.21) (Table 3).

**Table 3:** Moderation analysis: participant characteristics influencing SSB selection pre- and post-intervention

Participant characteristics	Pre-intervention N (%) (n = 33)	Post-intervention N (%) (n = 15)	Chi-Square	*p*-value
**Age (years)**				
18–39	24 (72.72)	12 (80.00)	0.29	0.58
40 +	9 (27.27)	3 (20.00)		
**Gender**				
Male	15 (45.45)	7 (46.66)	0.006	0.93
Female	18 (54.54)	8 (53.33)		
**Education**				
Less than high school	25 (75.75)	10 (66.66)	0.43	0.51
Intermediate or more	8 (24.24)	5 (33.33)		
**Monthly household income**				
< 68,961	26 (78.78)	9 (60.00)	1.84	0.17
≥ 68,961	7 (21.21)	6 (40.00)		
**Nutrition Facts Panel use**				
Never, rarely, sometimes	32 (96.96)	15 (100.00)	1	0.57*
Often, all the time	1 (3.03)	0 (0.00)		
**Frequency of needing help reading medical information**				
Never	28 (84.84)	14 (93.33)	0.67	0.41
Sometimes, often, always	5 (15.15)	1 (6.66)		
**Age of child the parent shopped for (years)**				
< 5	10 (30.30)	3 (20.00)	0.55	0.45
≥ 5	23 (69.69)	12 (80.00)		
**Gender of the child the parent shopped for**				
Boy	22 (66.66)	9 (60.00)	0.20	0.65
Girl	11 (33.33)	6 (40.00)		
**Child’s consumption of SSBs**				
Below sample median	10 (30.30)	2 (13.33)	1.58	0.21
At or above the sample median	23 (69.69)	13 (86.66)		

SSBs: Sugar-Sweetened Beverages

* Fischer Exact Test

NB: Table 3 presents interaction analyses testing whether participant characteristics moderated the intervention's effect on SSB purchasing

When stratified by child’s baseline SSB consumption (below vs. at/above the sample median), the intervention led to reductions in SSB purchasing across both groups. No statistically significant interaction was observed (*p* = 0.21), suggesting that MCIs were broadly effective regardless of prior child consumption levels.

To ensure demographic comparability between the pre- and post-intervention samples, Appendix Table A1 summarizes key socio-demographic characteristics. No statistically significant differences were observed between groups, confirming consistency and minimizing potential confounding bias.

## Discussion

This stepped-wedge randomized controlled study provides insights into the effectiveness of a multi-component public health intervention targeting parental behavior around SSB consumption.

The results are compelling, demonstrating a marked reduction in SSB purchases following the implementation of MCIs. Specifically, the odds of purchasing an SSB post-intervention decreased to 0.27, highlighting the protective effect of MCIs against SSB purchases.

These findings both corroborate and extend previous research in the field. A notable study by Campos et al. [25], conducted in a controlled convenience store laboratory setting, showed significant decreases in purchases of fruit drinks, sodas, and flavored water when PHWs were applied. Our research takes this a step further by demonstrating similar effects in real-world urban slum settings, emphasizing the practical applicability of MCIs in discouraging SSB purchases for children in diverse environments.

A particularly encouraging aspect of our findings is the consistent impact of MCIss across various educational and income levels. This suggests that MCIs can be an effective intervention even among low socioeconomic groups, potentially addressing health disparities related to SSB consumption. However, it’s worth noting that these results contrast with those of Mantzari et al. [26], who found PHWs less successful in reducing SSB selection in a lab-based study. This discrepancy underscores the importance of conducting real-world studies to accurately assess intervention effectiveness, as laboratory conditions may not always reflect the complexities of actual consumer behavior.

Our study also contributes to the growing body of evidence supporting the superiority of MCIs over other forms of health warnings. We found that MCIs provoked greater consideration of SSB-related harms among parents, indicating that creative visual representations can effectively communicate the adverse effects of excessive SSB consumption. This approach draws inspiration from successful implementations in other public health domains, particularly tobacco control [27, 28], where graphic warnings have been shown to significantly impact consumer behavior.

Importantly, our study builds on prior evidence demonstrating the added value of combining warning labels with educational or behavioral components. For example, Grummon et al. [8] reported that pairing pictorial warnings with social norm messaging enhanced reductions in sugary drink purchases compared to warnings alone. Similarly, Campos et al. [25] noted that supplemental materials improved attention to health messages and behavioral outcomes. These findings suggest that MCIs, such as ours—which integrated visual warnings, counseling, and educational pamphlets—may amplify individual components’ effects by reinforcing messages through diverse channels. This multi-pronged approach is especially relevant in low-literacy or resource-constrained populations, where single-mode communication may be insufficient.

The effectiveness of MCIs in our study was further evidenced by increased label noticeability and stronger label reactions, including heightened risk awareness and negative emotional responses. These findings align with those of Sillero-Rejon et al. [29], who observed increased avoidance and reaction to severe warnings in a laboratory experiment among drinkers. The emotional impact of MCIs appears to be a key factor in their effectiveness, potentially triggering a more visceral response than text-based warnings alone.

Beyond the immediate impact on purchasing behavior, our study revealed promising secondary outcomes. Notably, 53% of participants reported feeling more in control of making healthy eating decisions, and 45% expressed support for legislation requiring MCIs on SSBs. These findings suggest that MCIs could serve as an effective means of not only influencing individual behavior but also building public support for broader policy interventions aimed at promoting healthy food consumption and restricting access to less healthy options [5].

From a policy perspective, a national-level rollout of MCIs should ideally be integrated with mass media campaigns and community-based educational outreach, similar to tobacco control policies. Our findings suggest that combining visual cues with direct educational engagement could enhance message retention and behavioral change.

However, the implementation of MCIs is not without challenges. Issues surrounding label design and public acceptability remain significant considerations. Pechey et al. [14] found that while health warning labels depicting colon cancer generated the strongest adverse emotional reactions, they also had the lowest public acceptance. This highlights the delicate balance required in designing MCIs that are both effective and palatable to the public. Future research should focus on optimizing this balance to maximize the impact of MCIs while maintaining public support.

It’s also crucial to consider potential unintended consequences of implementing MCIs on SSBs. One possibility is a shift in consumer behavior towards artificially sweetened beverages, which, while lower in calories, may present their own health concerns. Additionally, previous studies have raised concerns about MCIs potentially triggering anger [30] or exacerbating weight bias [31]. These issues warrant careful investigation, particularly among diverse populations and individuals with mental health concerns or eating disorders. A comprehensive approach to SSB regulation should consider these potential side effects and incorporate strategies to mitigate them.

### Strengths and limitations

Our study benefits from several key strengths. The stepped-wedge randomized control design provides a robust framework for assessing the intervention’s effectiveness. The diverse sample of SSBs included in the study enhances the generalizability of our findings. Moreover, the real-life setting of urban slums offers valuable insights into the intervention’s effectiveness in challenging environments where SSB consumption may be particularly problematic. The consideration of socioeconomic factors in our analysis provides a nuanced understanding of the intervention’s impact across different population segments. Additionally, the two-week continuous MCI in each slum sector allowed sufficient time to assess its short-term effectiveness.

However, we must also acknowledge several limitations. There is a potential for participant behavior change due to awareness of the study’s objectives, a phenomenon known as the Hawthorne effect. This could lead to an overestimation of the intervention’s effectiveness. Our reliance on participant recall for SSBs purchased outside the study area introduces the possibility of recall bias, potentially affecting the accuracy of our data. Furthermore, the study’s timeframe limits our ability to assess the long-term effects of MCIs beyond the immediate intervention period. It remains unclear whether the observed changes in purchasing behavior would persist over extended periods or if consumers might become desensitized to the warnings over time. Additionally, the findings of this study may not be generalizable to broader or rural populations beyond urban slums. Pictorial health warnings could differentially impact segments of the population depending on literacy levels, health awareness, or economic status, potentially exacerbating existing health disparities if not implemented alongside tailored education efforts.

A key limitation of our study is the inability to isolate the specific effects of each component of the MCIs. While the combined approach was effective, we cannot determine whether the behavioral changes were primarily driven by PHWs, the counseling sessions, or the pamphlets. This calls for future studies using factorial designs to disentangle these effects and identify the most influential elements. Nonetheless, our findings align with existing research suggesting that integrated communication strategies may be more impactful than single interventions alone.

Additionally, reliance on self-reported purchasing behavior may be subject to social desirability bias, especially given the direct counseling component. Parents might underreport SSB purchases to align with perceived study expectations. Although weekly data collection minimized recall bias, future work should incorporate objective purchase measures or store-level sales data.

Our study provides compelling evidence for the effectiveness of MCIs in reducing SSB purchases among parents in urban slum settings. The consistency of this effect across socioeconomic groups and the positive secondary outcomes observed suggest that MCIs could be a valuable tool in public health efforts to reduce SSB consumption. However, careful consideration must be given to label design, potential unintended consequences, and long-term effectiveness. Further large-scale field studies are needed to assess the impact of PHW labels on SSBs in real-life settings over extended periods, ideally incorporating objective measures of SSB consumption and health outcomes. Such research will be crucial in informing evidence-based policies to combat the growing public health challenge of excessive SSB consumption.

## Conclusion

This study provides compelling evidence that MCIs on SSBs can significantly impact healthy decision-making by increasing awareness of added sugar content and risk perception. The MCI intervention effectively reduced SSB purchase frequency and increased parental intention to limit SSB consumption by their children. The consistent effectiveness of MCIs across demographic categories suggests their potential as a feasible and effective population-level intervention. Based on these findings, we recommend considering legislation requiring MCIs on SSBs to promote public health.

## Electronic supplementary material

Below is the link to the electronic supplementary material.

Supplementary Material 1

## Data Availability

Datasets generated during and/or analyzed during the current study are available from the corresponding author on reasonable request.
