# Supplementary material for: Effectiveness of a multi-component intervention including pictorial warnings to reduce sugar-sweetened beverage consumption - a randomized controlled trial
Source: Int J Behav Nutr Phys Act. 2025 Aug 30;22:115. doi: 10.1186/s12966-025-01800-0 (PMC12398057; doi:10.1186/s12966-025-01800-0)
Supplement: Supplementary file 1 — Supplementary Material 1 [file 12966_2025_1800_MOESM1_ESM.docx]

****Appendix Table A1.** Comparison of socio-demographic characteristics between pre- and post-intervention participants.**

| ****Variable**** | ****Pre-Intervention (n=33)**** | ****Post-Intervention (n=27)**** | ****p-value**** |
| --- | --- | --- | --- |
| Age (mean ± SD) | 36.3 ± 3.6 | 36.1 ± 3.8 | 0.87 |
| Gender (% Female) | 54.5% | 55.6% | 0.91 |
| Education (% ≥ High School) | 48.5% | 50.0% | 0.60 |
| Monthly Income (% ≥ ₹68,961) | 21.2% | 25.9% | 0.42 |
| Child Age (% ≥5 years) | 69.7% | 70.4% | 0.58 |
| Child Gender (% Boy) | 66.7% | 63.0% | 0.73 |

No statistically significant differences were found, indicating sample comparability across phases.
